# Emerging themes in microbial stress response and mechanistic insights: key findings from the fall 2024 ASM Theobald Smith Society meeting

**DOI:** 10.1128/msphere.01008-24

**Published:** 2025-01-14

**Authors:** Duhita Sant, Akilah I. Mateen, Raymond F. Sullivan, Jeffrey M. Boyd, Valerie J. Carabetta, Srujana S. Yadavalli, Jennifer S. Sun

**Affiliations:** 1 Center for Advanced Biotechnology and Medicine, Rutgers University, Piscataway, New Jersey, USA; 2 Department of Chemistry and Biochemistry, Seton Hall University, South Orange, New Jersey, USA; 3 Department of Marine and Coastal Sciences, Rutgers University, New Brunswick, New Jersey, USA; 4 Department of Biochemistry and Microbiology, Rutgers University, New Brunswick, New Jersey, USA; 5 Cooper Medical School, Rowan University, Camden, New Jersey, USA; 6 Department of Genetics, Rutgers University, Piscataway, New Jersey, USA; 7 Waksman Institute of Microbiology, Rutgers University, Piscataway, New Jersey, USA; University of Michigan, Ann Arbor, Michigan, USA

**Keywords:** branch meeting, microbiology, environmental biology, infectious diseases, clinical microbiology, applied microbiology, cellular biology, molecular biology

## Abstract

The annual fall meeting for the Theobald Smith Society was held in November 2024 on the campus of Rutgers University—New Brunswick. Eighty-six branch members from across New Jersey attended the meeting, composed of undergraduate, graduate, and postdoctoral trainees, faculty members, and government and industry professionals. This report highlights the breadth and diversity of research conducted by American Society for Microbiology members in the Theobald Smith Society and celebrates their groundbreaking discoveries.

## MEETING SUMMARY INFORMATION

**Fig 1:**
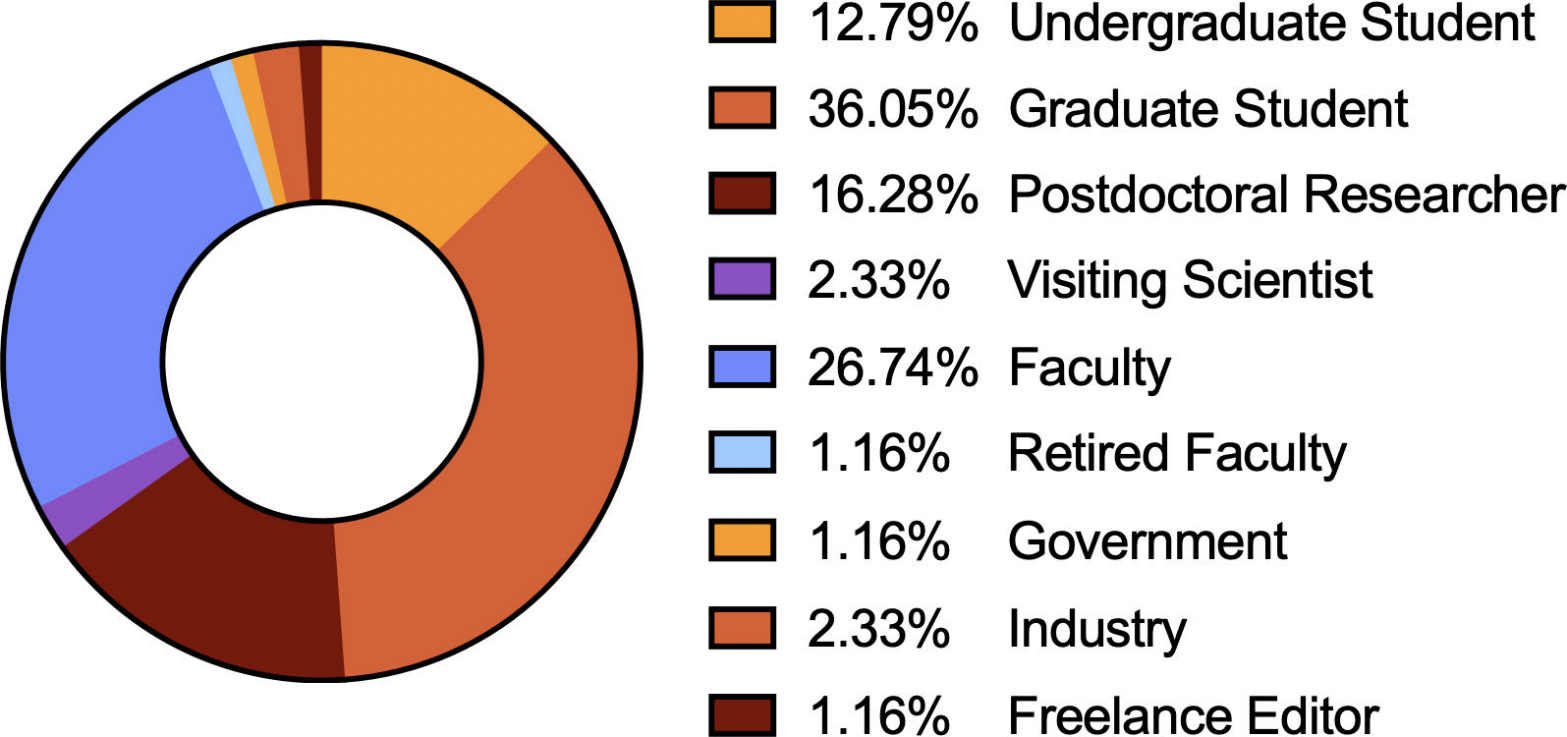
Distribution of participants’ career stages (86 total attendees), demonstrating overwhelming trainee participation at the Theobald Smith Society’s Fall 2024 Symposium.

**Fig 2:**
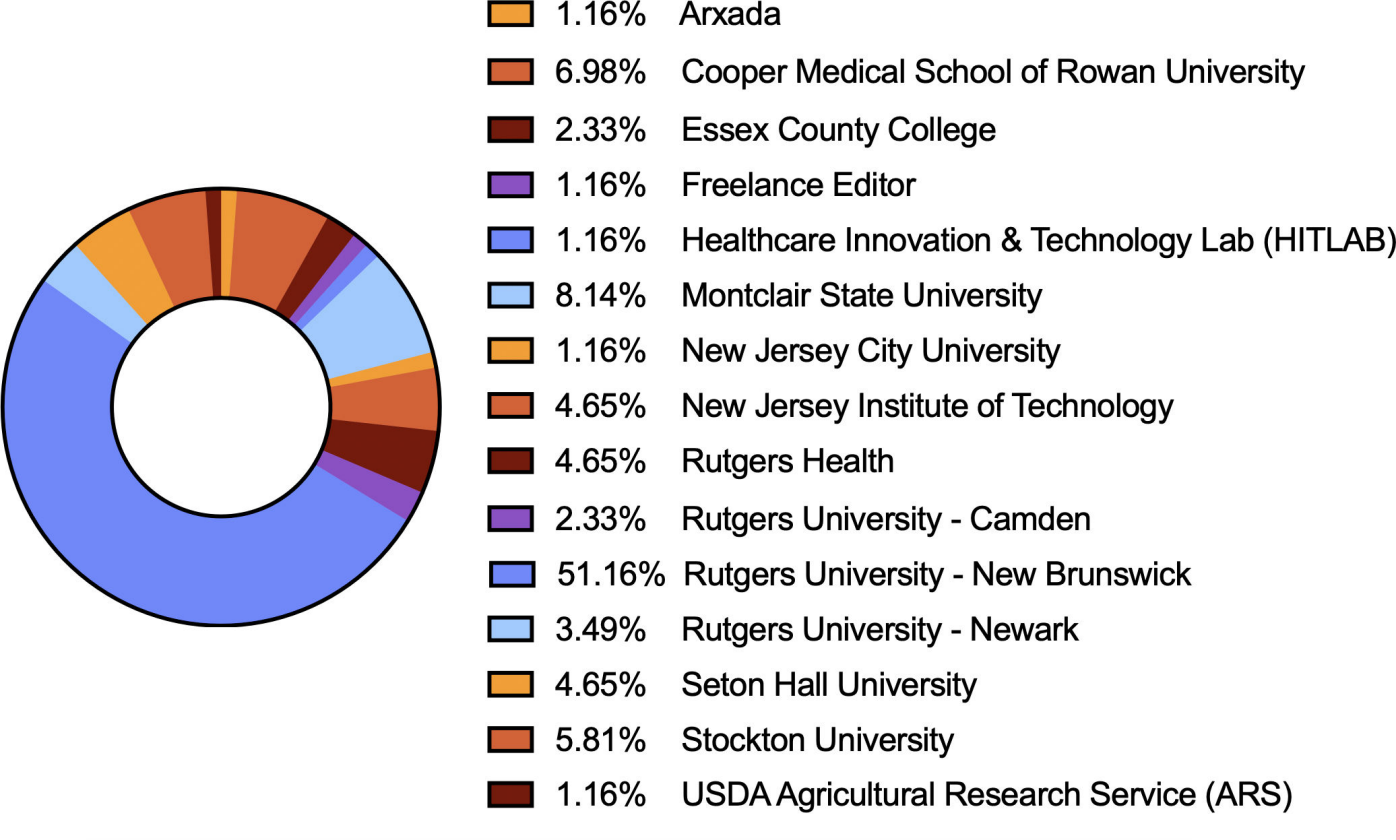
Distribution of institutions represented at the Theobald Smith Society’s Fall 2024 Symposium (86 total attendees).

**Fig 3:**
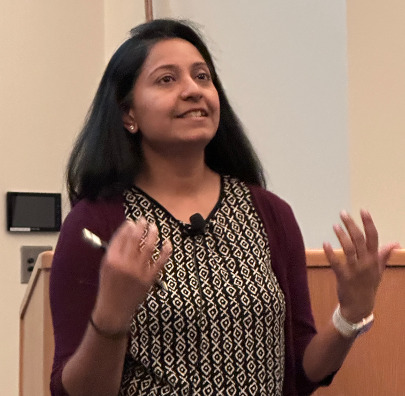
2024 Young Investigator Awardee Srujana S. Yadavalli delivered a lecture on “Translational Profiling of Stress-Induced Small Proteins Uncovers a Connection between Distinct Signaling Systems.”

**Fig 4:**
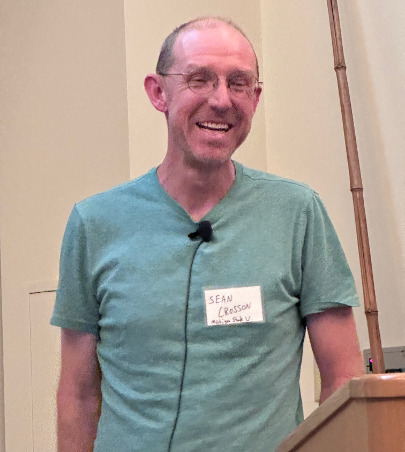
Dr. Sean Crosson, Michigan State University.

**Fig 5:**
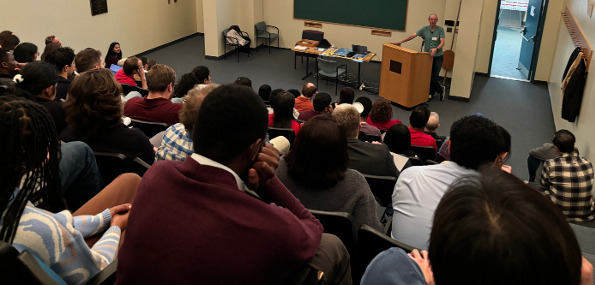
Dr. Sean Crosson discussing “Molecular Mechanisms of Environmental Stress Resistance in Bacteria.”

**Fig 6:**
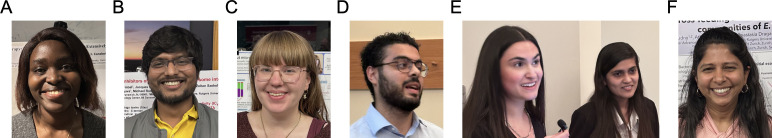
Six invited speakers presented 10 minute lectures describing their ongoing research efforts. Speakers included (**A**) Precious Newman, Cooper Medical School of Rowan University; (**B**) Dr. Arkajyoti Dutta, Rutgers University; (**C**) Dr. Liisa Veerus, Rutgers University; (**D**) Amir George, Rutgers Health; (**E**) Naima Zaheer and Sarah Krisak, Montclair State University; and (**F**) Dr. Duhita G. Sant, Rutgers University.

**Fig 7:**
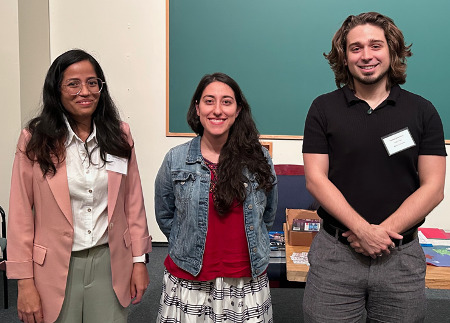
Poster research presentation awardees (left to right): Devi Kumari Dhakal Guadel, New Jersey Institute of Technology; Irene Goldwasser, Rutgers University; and Luke Foster, Rutgers University. Each winner received monetary awards sponsored by the Theobald Smith Society and 2025 ASM memberships sponsored by Dr. Liisa Veerus, 2024 U.S. Young Ambassador for the New Jersey area.

The annual fall meeting for the Theobald Smith Society, New Jersey Branch of the American Society for Microbiology (ASM), was held in November 2024 at the Institute of Marine and Coastal Sciences at Rutgers, The State University of New Jersey, in New Brunswick, NJ. The branch includes members across all of New Jersey. The majority of the 86 attendees were trainees (12.79% undergraduate students, 36.05% graduate students, and 16.28% postdoctoral researchers), joined by faculty members and government and industry professionals (Fig. 1). Regional institutions were well represented (Fig. 2). 2024 Theobald Smith Society Young Investigator Award Recipient Dr. Srujana Samhita Yadavalli (Rutgers University—New Brunswick) presented the 2024 Young Investigator Lecture, “Translational Profiling of Stress-Induced Small Proteins Uncovers a Connection between Distinct Signaling Systems” (Fig. 3). ASM Distinguished Lecturer Dr. Sean Crosson (Michigan State University) presented “Molecular Mechanisms of Environmental Stress Resistance in Bacteria” (Fig. 4 and 5). Thirty-six poster presenters from nine distinct undergraduate, professional, and/or research institutions within the branch boundaries contributed abstracts to the meeting, including Cooper Medical School of Rowan University, Montclair State University, New Jersey Institute of Technology, Rutgers University—Newark, Rutgers Health, Rutgers University—Camden, Rutgers University—New Brunswick, Seton Hall University, and Stockton University. Six trainees were selected to present 10 minute lectures describing their ongoing research endeavors (Fig. 6). The meeting concluded with a poster research award presentation (Fig. 7) and forward-thinking statements in anticipation of the annual spring meeting for the Theobald Smith Society and ASM Microbe 2025.

## KEYNOTE ADDRESS: YOUNG INVESTIGATOR LECTURE

The first keynote address was given by Srujana Samhita Yadavalli, Ph.D., recipient of the 2024 Young Investigator Award from the Theobald Smith Society (Fig. 3). Her lab in the Waksman Institute of Microbiology and the Department of Genetics at Rutgers University–New Brunswick focuses on identifying and characterizing small proteins, typically containing up to 50 amino acids in prokaryotes (or 100 amino acids in eukaryotes). Due to their ubiquity in critical cellular processes, such as transport and signal transduction, and their emerging role in gene expression, small proteins can be considered a “Swiss Army knife” group of regulators (1). Their size presents a challenge for functional analysis using traditional techniques like SDS-PAGE, which warrants the development of new analytical tools. The conditional expression of small proteins is also of great interest in microbial research, as their responses to stress can reveal significant insights into their biological roles and understanding of antimicrobial resistance.

Using translation initiating profiling, Dr. Yadavalli’s lab identified 17 whose expression is upregulated in low Mg^2+^ conditions. Many of these small proteins were found to be membrane bound, where they can interact with larger membrane proteins/complexes such as two-component signaling systems. The expression of 11 of the identified small proteins is regulated by the PhoQ-PhoP signaling system. One outlier, 34-amino-acid-long YoaI, which was previously undetected in other stressful conditions (e.g., heat shock), is transcriptionally regulated by the PhoR-PhoB system, but its translation is higher in low Mg^2+^, making it detectable under these conditions. Additionally, YoaI is responsible for activating the EnvZ-OmpR system, making it a connector of two distinct signaling systems: PhoR-PhoB and EnvZ-OmpR. These results emphasize the necessity to investigate more comprehensively the roles of small proteins and the connections between different regulatory pathways. A preprint of the work has recently been submitted (2).

## KEYNOTE ADDRESS: ASM DISTINGUISHED LECTURE

Sean Crosson, Ph.D., the Professor Rudolph Hugh Endowed Chair at Michigan State University, delivered the second keynote address (Fig. 4 and 5). His research is focused on understanding molecular mechanisms that underlie the ability of bacterial cells to adapt to complex, dynamic environments, including mammalian hosts. In the first portion of his presentation, Dr. Crosson talked about *Caulobacter crescentus*, a model organism that exhibits unique polar cell development, allowing it to mediate attachment to various surfaces, including abiotic and biotic interfaces, self-surfaces, and air-liquid boundaries. This attachment is facilitated by Wzy-dependent polysaccharides, which contribute to the formation of a holdfast—a specialized adhesive structure (3). Dr. Crosson’s lab utilized barcoded mutants to calculate fitness, focusing mainly on enriching mutants that cannot adhere to surfaces like cheesecloth. This approach led to identifying potential holdfast biosynthesis genes, including *ccna_02722*, which encodes a protein that helps cells attach but does not allow them to adhere to cheesecloth (4). They further investigated the holdfast biosynthesis pathway, revealing that the holdfast polysaccharide consists of common sugars, including glucose and mannose. Having identified key enzymes and the sugars involved, they hope to reconstitute the holdfast biosynthesis pathway *in vitro* to better understand its composition and assembly.

In the second portion of the presentation, Dr. Crosson presented their most recent research findings on *Bacteroides fragilis*, a key player in colitis and pouchitis, a condition that often arises following the surgical creation of a J-pouch after colon removal. By collaborating with clinicians and ecologists, Dr. Crosson’s lab found that the treatment of *B. fragilis* with physiologically relevant concentrations of the secondary bile acid deoxycholate reduced cell growth and remodeled transcription of one-quarter of the bacterium’s genome, resulting in the discovery of novel bile resistance genes in *B. fragilis*. They also discovered the mechanisms behind resistance and the survival of *B. fragilis* inside the J-pouch. He hopes their findings will contribute to understanding the causes of inflammatory bowel diseases (5).

## PRESENTATION THEMES

**Fig 8:**
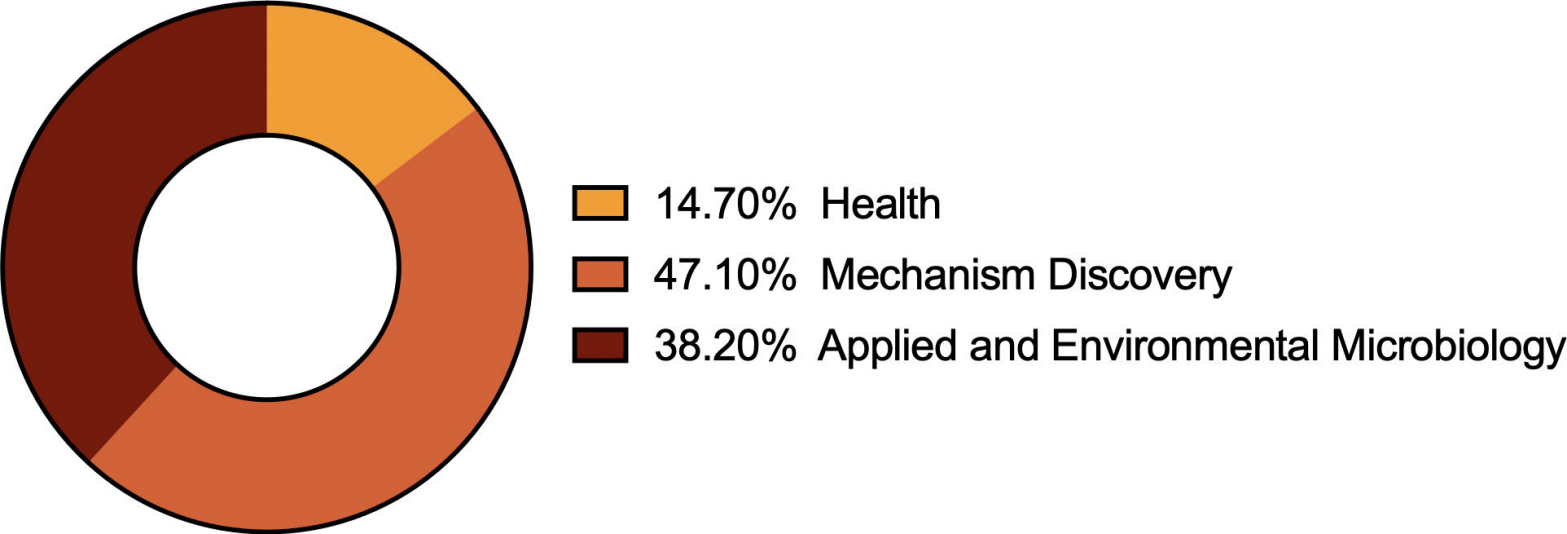
Distribution of topical areas for submitted abstracts (36 total presentations).

The oral (Fig. 6) and poster (Fig. 7) presentations were grouped into the three scientific units established by ASM this year: Health, Hechanism Discovery, and Applied and Environmental Microbiology (Fig. 8).

### ASM Health (includes phage therapy, drug discovery, antimicrobial resistance, and microbiome)

Health included topics such as phage therapy, drug discovery, antimicrobial resistance, and microbiome. Within each subtopic, trainees presented ideas covering microbial community shifts in Arctic and Antarctic soils under warming, quantifying drug uptake and metabolites within *Mycobacterium smegmatis* as well as from Gram-negative bacteria, offering a valuable tool for antitubercular and antibacterial drug development.

One of the oral presentations evaluated how breast milk oligosaccharide composition is influenced by the gestational gut microbiome, which highlighted the interplay between maternal gut microbiota and breast milk composition, shedding light on mechanisms supporting the transgenerational inheritance of a healthy microbiome. Another oral presentation discussed the potential of combining bacteriophage therapy with antibiotics, such as ceftazidime and meropenem, to combat extensively drug-resistant *Acinetobacter baumannii*, showing promising synergy in reducing bacterial growth rates and improving antibiotic efficacy. Ongoing research in phage therapy, drug discovery, antimicrobial resistance, and microbiome by members of the New Jersey Branch advances our understanding of microbes and human health.

### ASM Mechanism Discovery (includes microbial physiology and metabolism)

Mechanism Discovery was the largest category. It included broader disciplines like systems biology, molecular genetics, protein structure-function studies, cellular physiology, metabolism, and microbial evolution. Poster presentations spanned research on diverse physiology and metabolism discoveries under various conditions, including the effects of various small proteins on bacterial metabolism, identifying how the spatial organization of enzymes follow a unique sphingolipid synthesis pathway, testing the impact of colimitation on bacterial susceptibility to antibiotics, and predicting microbial evolution under various stress environments including climate stress. Ongoing research in microbial physiology and metabolism at the New Jersey Branch institutions will enhance our understanding of how microbes behave, adapt, and survive in various environmental conditions; the mechanisms by which microbes cause disease; and how these processes can be harnessed to improve the world around us.

One oral presentation from this theme identified the P-stalk binding site of the ricin A subunit as a novel target for developing allosteric inhibitors against ricin and Shiga toxins. Considering no adequate therapy exists for ricin and Shiga toxin intoxication, this can be a promising lead for drug discovery in the future. Another oral presentation from this category discussed the effects of cross-feeding interactions in microbial communities on microbial adaptation, which was studied using barcoded transposon mutant libraries of *Escherichia coli* auxotrophs. The study revealed how these interactions reshape the distribution of fitness effects, reduced deleterious mutations, increased beneficial ones, and reversed selection on specific mutants.

### ASM Applied and Environmental Microbiology

Research relating to environmental microbiology and microbial ecology was prominent. One oral presentation investigated the ability of artificial root exudates as a microbial food source to remediate the deleterious effects of heavy metal contamination in soil (Liberty State Park, Jersey City, NJ), which could serve as an eco-friendly replacement for traditional fertilizers. By measuring phosphatase activity and soil respiration rate, a balanced combination of specific biomolecules, like carbohydrates, amino acids, and organic acids, increased the number of microbes contributing to soil viability.

Several poster presentations similarly focused on the symbiotic relationship between microbe diversity in soil and plant health, ranging from studies on the effects of temperature changes on microbial community composition in the Arctic and Antarctic regions to identifying potential sharing patterns of insecticidal bacteria among different crops. Three students were awarded prizes for their posters covering research relating to this theme (Fig. 7). This work included discovering a thermophilic gammaproteobacterium member of the Chromatiales family from a hydrothermal vent on the East Pacific Rise; bioremediation of 1,4-dioxane using a novel bacterium, *Azoarcus* sp. DD4, with 1-propanol and propane as carbon sources; and exploration of the viability of soundproof panels by functionalizing secondhand cotton materials with fungi.

## CONCLUSIONS

The fall 2024 symposium of the Theobald Smith Society highlighted many pressing areas of concern to researchers, particularly how microbes adapt to changes in their immediate cellular environment and from external stressors. The research reflects the growing interdisciplinarity required to gain substantial insights into the microbial landscape. For meeting details, including all abstracts, go to https://www.njmicrobe.org/fall-2024-symposium.

## References

[1] Yadavalli SS, Yuan J. 2022. Bacterial small membrane proteins: the swiss army knife of regulators at the lipid bilayer. J Bacteriol 204:e00344-21. doi:10.1128/JB.00344-21 PMC8765417 34516282

[2] Vellappan S, Sun J, Favate J, Jagadeesan P, Cerda D, Shah P, Yadavalli SS. 2024. Translation profiling of stress-induced small proteins reveals a novel link among signaling systems. bioRxiv:2024.09.13.612970. doi:10.1101/2024.09.13.612970

[3] Hershey DM, Porfírio S, Black I, Jaehrig B, Heiss C, Azadi P, Fiebig A, Crosson S. 2019. Composition of the holdfast polysaccharide from Caulobacter crescentus. J Bacteriol 201:e00276-19. doi:10.1128/JB.00276-19 31209074 PMC6689307

[4] Hershey DM, Fiebig A, Crosson S. 2019. A genome-wide analysis of adhesion in Caulobacter crescentus identifies new regulatory and biosynthetic components for holdfast assembly. mBio 10:e02273-18. doi:10.1128/mBio.02273-18 30755507 PMC6372794

[5] Fiebig A, Schnizlein MK, Pena-Rivera S, Trigodet F, Dubey AA, Hennessy MK, Basu A, Pott S, Dalal S, Rubin D, Sogin ML, Eren AM, Chang EB, Crosson S. 2024. Bile acid fitness determinants of a Bacteroides fragilis isolate from a human pouchitis patient. mBio 15:e0283023. doi:10.1128/mbio.02830-23 38063424 PMC10790697

